# Full-Lung Prophylaxis against SARS-CoV-2 by One-Shot or Booster Intranasal Lentiviral Vaccination in Syrian Golden Hamsters

**DOI:** 10.3390/vaccines11010012

**Published:** 2022-12-21

**Authors:** Benjamin Vesin, Pierre Authié, Catherine Blanc, Ingrid Fert, Amandine Noirat, Fabien Le Chevalier, Yu Wei, Min-Wen Ku, Kirill Nemirov, François Anna, David Hardy, Cyril Planchais, Hugo Mouquet, Françoise Guinet, Pierre Charneau, Laleh Majlessi, Maryline Bourgine

**Affiliations:** 1 TheraVectys Joint Lab, Institut Pasteur, Université Paris Cité, F-75015 Paris, France; 2 Histopathology Platform, Institut Pasteur, Université Paris Cité, F-75015 Paris, France; 3 Laboratory of Humoral Immunology, Institut Pasteur, Université Paris Cité, F-75015 Paris, France; 4 Lymphocytes and Immunity Unit, Institut Pasteur, Université Paris Cité, F-75015 Paris, France

**Keywords:** lentiviral vectors, SARS-CoV-2 variants, intranasal vaccination, mucosal immunity, lung inflammation, cross-neutralization

## Abstract

Following the breakthrough of numerous severe acute respiratory syndrome coronavirus 2 (SARS-CoV-2) variants in recent months and the incomplete efficiency of the currently available vaccines, development of more effective vaccines is desirable. Non-integrative, non-cytopathic and non-inflammatory lentiviral vectors elicit sterilizing prophylaxis against SARS-CoV-2 in preclinical animal models and are particularly suitable for mucosal vaccination, which is acknowledged as the most effective in reducing viral transmission. Here, we demonstrate that a single intranasal administration of a vaccinal lentiviral vector encoding a stabilized form of the original SARS-CoV-2 Spike glycoprotein induces full-lung protection of respiratory tracts and strongly reduces pulmonary inflammation in the susceptible Syrian golden hamster model against the prototype SARS-CoV-2. In addition, we show that a lentiviral vector encoding stabilized Spike of SARS-CoV-2 Beta variant (LV::S_Beta-2P_) prevents pathology and reduces infectious viral loads in lungs and nasal turbinates following inoculation with the SARS-CoV-2 Omicron variant. Importantly, an intranasal boost with LV::S_Beta-2P_ improves cross-seroneutralization much better in LV::S_Beta-2P_-primed hamsters than in their counterparts primed with an LV-encoding Spike from the ancestral SARS-CoV-2. These results strongly suggest that an immune imprint with the original Spike sequence has a negative impact on cross-protection against new variants. Our results tackle the issue of vaccine effectiveness in people who have already been vaccinated and have vanished immunity and indicate the efficiency of LV-based intranasal vaccination, either as a single dose or as booster.

## 1. Introduction

Numerous prophylactic vaccine platforms have been developed to fight the severe acute respiratory syndrome coronavirus-2 (SARS-CoV-2) that caused the worldwide pandemic coronavirus disease 2019 (COVID-19) [1]. The main authorized vaccines are based on the Spike glycoprotein (S) of SARS-CoV-2 (S_CoV-2_). They are approximately 80% effective in preventing SARS-CoV-2 infection by eliciting primarily humoral and—to a lesser extent—cellular immunity. However, recent epidemiological data have shown that vaccination does not prevent reinfection and that the highly protective benefit of full vaccination wanes rapidly, particularly against the ceaselessly emerging SARS-CoV-2 Variants of Concern (VoCs), such as Omicron sub-variants [2,3]. The immunity generated by intramuscularly administered vaccines seems insufficient against respiratory viruses, including SARS-CoV-2, highlighting the need for alternative vaccine platforms. Intranasal (i.n.) vaccination can induce not only serum antigen-specific IgG but also antigen-specific IgA in the lower and upper respiratory tract [4]. Secretory IgA antibodies, present in polymeric forms at mucosal surfaces, display stronger virus-binding/neutralizing activities than plasma IgG or IgA that are primarily present as monomers [5,6]. In addition, resident memory lymphocytes at the respiratory mucosa can largely contribute to the protection of airways and help in reducing the transmission chain [7]. Thus, vaccines administered by the i.n. route can provide a superior and better targeted immunity against SARS-CoV-2.

Lentiviral vectors (LV) have been extensively used in gene therapy and transgenesis, based on their remarkable efficiency at transducing a wide range of non-dividing as well as dividing cells [8]. Substitution of their viral envelope protein with the heterologous glycoprotein of the vesicular stomatitis virus, to which human populations have negligible exposure, made it possible to use them as vaccinal vectors, without particular anti-vector immunity [9]. Integrative and non-integrative forms of LV can carry large and complex transgenes and induce both strong and long-lasting cellular and humoral responses [10]. We previously described the efficiency of LV-based vaccine candidates in protection against several infectious diseases, including malaria, Zika virus disease, and tuberculosis in preclinical models [11,12,13]. Only non-integrative forms of LV will be used in vaccination. We have recently shown that systemic immunization with an LV encoding the full-length membrane anchored S sequence from the ancestral WA1 SARS-CoV-2 (S_WA1_) induced high serum neutralizing activity in rodents but conferred only partial protection against SARS-CoV-2 [14]. In contrast, we obtained sterilizing protection when an i.n. boost was given after systemic prime injection [14,15]. An i.n. boost also protects the central nervous system in the recently generated B6.K18-hACE2^IP-THV^ transgenic mice expressing the human Angiotensin-Converting Enzyme 2 (ACE2) and displaying strong permissiveness of the lung and brain to SARS-CoV-2 replication [15].

Syrian golden hamsters (*Mesocricetus auratus*) are naturally permissive to SARS-CoV-2 replication because of the high ACE2 sequence homology between human and hamster [16]. Following the binding of the Receptor-Binding Domain (RBD) of SARS-CoV-2 to ACE2, a productive infection and COVID-19-like manifestations are observed in these animals with similar pathological changes to those seen in lung tissues from COVID-19 patients [16]. In the present study, we designed a series of non-integrative LV vaccine candidates that express various forms of S_CoV-2_ and assessed their immunogenicity and cross-protective efficiency against SARS-CoV-2 challenge after administration of a single i.n. injection in hamsters. We demonstrated that i.n. administration of an LV-encoding S_WA1_ was as protective as an intramuscular (i.m.) prime and i.n. boost regimen, conferring sterilizing pulmonary protection against homologous SARS-CoV-2 challenge. Moreover, we showed that i.n. vaccination was able to reduce disease severity and viral loads following inoculation of SARS-CoV-2 Omicron BA.1 sub-variant. In addition, we showed that boosting with an LV encoding a S_Beta_ can broaden the cross sero-neutralization and cross-protection properties against SARS-CoV-2 VoCs. We also provided evidence of a negative impact of a humoral immune imprint in hamsters primed with the initial S_WA1_ sequence, which is much less receptive to a boost with an adapted S sequence from new variants. Our results indicate that i.n. vaccination with LV is an effective and promising strategy to elicit protective immunity against SARS-CoV-2 VoCs.

## 2. Materials and Methods

### 2.1. Construction and Production of LV Expressing S Protein

Construction of LV::S_WA1_ and LV::S_WA1_-_ΔF2P_ was described elsewhere [14,15]. Briefly, codon-optimized S sequences (1-1262) from the ancestral S_WA1_ and Beta SARS-CoV-2 strains were amplified and inserted into the pFlap lentiviral plasmid by restriction/ligation between BamHI and XhoI sites, between the native human ieCMV promoter and a mutated Woodchuck Posttranscriptional Regulatory Element (WPRE) sequence. A directed mutagenesis was performed by use of a Takara In-Fusion kit to introduce the 2 proline mutations in S_WA1_ or S_Beta_, on the corresponding pFlap plasmids.

### 2.2. Production and Titration of LV

Lentiviral particles were produced by transient calcium phosphate co-transfection of HEK293T cells with the vector plasmids pFlap/S_Cov-2_, a vesicular stomatitis virus G Indiana envelope plasmid and an encapsidation plasmid pD64V for the production of integration-deficient vectors. Supernatants were harvested at 48 h post-transfection, clarified by 6-min centrifugation at 2500 rpm at 4 °C. LV were aliquoted and stored at −80 °C. Vector titers were determined by transducing HEK293T cells treated with aphidicolin. The titer, proportional to the efficacy of nuclear gene transfer, is determined as Transduction Unit (TU)/mL by qPCR on total lysates at day 3 post-transduction, by use of forward 5′-TGG AGG AGG AGA TAT GAG GG-3′ and reverse 5′-CTG CTG CAC TAT ACC AGA CA-3′ primers, specific to pFLAP plasmid and forward 5′-TCT CCT CTG ACT TCA ACA GC-3′ and reverse 5′-CCC TGC ACT TTT TAA GAG CC-3′ primers specific to the host housekeeping *gadph* gene, as previously described [17].

### 2.3. SDS-PAGE and Western Blotting

Six-well plates were seeded with HEK293T cells (2 10^6^ cells/well), and after overnight growth transduced with LV-encoding SARS-CoV-2 S transgenes at a multiplicity of infection of 10. Cell lysates were harvested 48 h post-transduction and quantified. After heating for 5 min at 95 °C bolt with Bolt sample buffer, samples were loaded on a precast Bolt 4–12% Bis-Tris gel (Invitrogen). Proteins were transferred to a nitrocellulose membrane using an iBlot2 dry blotting system (Invitrogen), and the membrane was blocked with TBST blocker (Tris-buffered saline (TBS) containing 0.2% Tween 20 and 5% milk). Following 1 h blocking, the membrane was incubated overnight with an anti-SARS-CoV-2 S2 rabbit polyclonal antibody (SinoBiological 40590-T62) in TBST blocker. The membrane was then washed three times with TBST for 10 min and subsequently incubated for 1 h with 1:2500 DyLight 800-conjugated goat anti-rabbit IgG (H+L) secondary antibody (Invitrogen, Cat # SA5-35571) in TBST Blocker. Finally, the membrane was washed three times with TBST for 10 min and developed using an ODYSSEY CLx Infrared Imaging System (Li-COR). E-PAGE SeeBlue Pre-stained Standard (Invitrogen, Waltham, MA, USA) was used as ladder.

### 2.4. Hamsters

Male *Mesocricetus auratus* Syrian golden hamsters (Janvier, Le Genest Saint Isle, France) were purchased mature and weighed between 80 and 100 gr at the beginning of the experiments. Hamsters were housed in individually ventilated cages under specific pathogen-free conditions during the immunization period. For SARS-CoV-2 infection they were transferred into individually filtered cages placed inside isolators in the animal facility of Institut Pasteur. Prior to i.m. or i.n. injections, hamsters were sedated with isoflurane inhalation or i.p. injection of Ketamine (Imalgene, 100 mg/kg) and Xylazine (Rompun, 5 mg/kg).

### 2.5. Ethical Approval of Animal Studies

Experimentation on hamsters was realized in accordance with the European and French guidelines (Directive 86/609/CEE and Decree 87-848 of 19 October 1987) subsequent to approval by the Institut Pasteur Safety, Animal Care and Use Committee, protocol agreement delivered by local ethical committee (CETEA #DAP200007) and Ministry of High Education and Research (APAFIS#24627-2020031117362508 v1).

### 2.6. Production of SARS-CoV-2 Spike Proteins

Codon-optimized nucleotide fragments encoding a stabilized version of SARS-CoV-2 WA1 or Omicron BA.1 spike (HexaPro) ectodomain (followed by a foldon trimerization motif) and WA1 or Omicron BA.1 RBD proteins containing C-terminal tags (Hisx8-tag, Strep-tag, and AviTag) were synthesized and cloned into pcDNA3.1/Zeo^(+)^ expression vector (Thermo Fisher Scientific). Recombinant proteins were produced by transient transfection of exponentially growing Freestyle 293-F suspension cells (Thermo Fisher Scientific, Waltham, MA, USA) using polyethylenimine (PEI) precipitation method as previously described (PMID: 25910833). Proteins were purified from culture supernatants by high-performance chromatography using the Ni Sepharose^®^ Excel Resin according to manufacturer’s instructions (GE Healthcare, Chicago, IL, USA), dialyzed against PBS using Slide-A-Lyzer^®^ dialysis cassettes (Thermo Fisher Scientific), quantified using NanoDrop 2000 instrument (Thermo Fisher Scientific), and controlled for purity by SDS-PAGE using NuPAGE 3–8% Tris-acetate gels (Life Technologies, Carlsbad, CA, USA), as previously described (PMID: 25910833).

### 2.7. Humoral Response

Immunoglobulin G (IgG) Abs were detected by an enzyme-linked immunosorbent assay (ELISA) by use of recombinant stabilized S_CoV-2_ and RBD proteins from the SARS-CoV-2 WA1 or Omicron strains. Nunc Polysorp ELISA plates (ThermoFisher, 475094) were coated at 1 µg/mL in 50 mM Na_2_CO_3_ pH 9.6 at 4 °C overnight. After incubation, plates were washed with 1 × PBS + 0.05% Tween-20 (PBST) and blocked with PBST + 1% BSA for 2 to 3 h at 37 °C. Plates were incubated with serial dilutions of sera in PBS-T + 1% BSA for 1.5 h at 37 °C. Following washes, rabbit anti-hamster IgG-horseradish peroxidase conjugate (Jackson Immuno Research, West Grove, PA, USA, M37470) was used as secondary Ab, and 3,5,3′5′-tetramethylbenzidine (Eurobio Scientific, Essonnes, France, 5120-0047) was used as the substrate to detect Ab responses. Reactions were stopped with 50 µL of 2 M sulfuric acid. Endpoint titers were calculated as the highest serum dilution that resulted in an absorbance value greater than that mean +3SD of pre-immune sera.

### 2.8. SARS-CoV-2 Inoculation

Hamsters were anesthetized by i.p. injection of a Ketamine and Xylazine mixture, transferred into a biosafety cabinet 3 and inoculated i.n. with 50 µL of viral inoculum containing 0.3 × 10^5^ TCID_50_ of the WA1 [18] or the Omicron BA.1 variant (Pango lineage BA.1, GISAID: EPI_ISL_6794907 and EPI_ISL_7413964) of SARS-CoV-2 clinical isolate [19]. Animals were housed in an isolator in BioSafety Level 3 animal facilities of Institut Pasteur. The organs recovered from the infected animals were manipulated according to the approved standard procedures of these facilities.

### 2.9. Pseudovirus Neutralization Assay

Nab quantification was assessed via an inhibition assay which uses HEK293T cells stably expressing human ACE2 (HEK 293T-ACE2) and non-replicative S_CoV-2_ pseudo-typed LV particles which harbor the reporter luciferase firefly gene, allowing quantitation of the host cell invasion by mimicking fusion step of native SARS-CoV-2 virus, as described elsewhere [6]. Serum samples or clarified lung homogenates were heat inactivated at 56 °C for 30 min. Serial four-fold dilutions of samples diluted in 25 µL DMEM-glutamax (Gibco, Illkirch, France, 21063-029) containing 10% heat-inactivated FCS, 100 U/mL penicillin and 100 mg/mL streptomycin and 1 mM sodium pyruvate (Gibco, 11360-070) were mixed with 1 ng of S_CoV-2_ pseudo-typed LV p24 equivalent in 25 µL for 30 min at room temperature, in U-bottom plates. Samples were then transferred into clear-flat-bottom 96-well-black-plates (Corning, NYC, NY, USA, CLS3603) containing 2 10^4^ HEK 293T-ACE2 cells. The plates were incubated for 72 h at 37 °C and then assayed for luciferase expression using the ONE-Glo™ Luciferase Assay System (Promega, Madison, WI, USA, E6120) on an EnSpire plate reader (PerkinElmer, Waltham, MA, USA). EC50 was reported as the reciprocal of the serum dilution conferring 50% of infection of HEK 293T-ACE2 cells by lentiviral vectors bearing the indicated S_CoV-2_ variants.

### 2.10. Determination of Viral Loads in the Organs

Lungs and nasal turbinates (NT) were removed aseptically and immediately frozen at −80 °C. RNA from circulating SARS-CoV-2 was prepared from lungs as recently described [14]. Briefly, lung homogenates were prepared by thawing and homogenizing of the organs using lysing matrix A (MP Biomedicals, Santa Ana, CA, USA, 116913050-CF) in 500 μL of ice-cold PBS in an MP Biomedical Fastprep 24 Tissue Homogenizer and were used to determine viral loads by E-specific qRT-PCR. Alternatively, total RNA was prepared from lungs or NT by addition of lysing matrix D (MP Biomedical, 116910050-CF) containing 1 mL of TRIzol reagent (ThermoFisher, 15596026) and homogenization at 30 s at 6.0 m/s twice using MP Biomedical Fastprep 24 Tissue Homogenizer. These RNA preparations were used to determine viral loads by Esg-specific qRT-PCR or inflammatory mediators.

SARS-CoV-2 E gene or E sub-genomic mRNA (Esg RNA) was quantitated following reverse transcription and real-time quantitative TaqMan^®^ PCR, using a SuperScript™ III Platinum™ One-Step qRT-PCR Kit (Invitrogen, 11732020) and specific primers and probe (Eurofins, Nantes, France) as previously described [20,21]. The standard curve of Esg mRNA assay was performed using in vitro transcribed RNA derived from PCR fragment of “T7 SARS-CoV-2 Esg mRNA”. The in vitro transcribed RNA was synthesized using the T7 RiboMAX Express Large-Scale RNA production system (Promega, P1320) and purified by phenol/chloroform extraction and two successive precipitations with isopropanol and ethanol. Concentration of RNA was determined by optical density measurement, diluted to 10^9^ genome equivalents/μL in RNAse-free water containing 100 μg/mL tRNA carrier, and stored at −80 °C. Serial dilutions of this in vitro transcribed RNA were prepared in RNAse-free water containing 10 μg/mL tRNA carrier to build a standard curve for each assay. PCR conditions were: (i) reverse transcription at 55 °C for 10 min, (ii) enzyme inactivation at 95 °C for 3 min, and (iii) 45 cycles of denaturation/amplification at 95 °C for 15 s, 58 °C for 30 s. PCR products were analyzed on an ABI 7500 Fast real-time PCR system (Applied Biosystems, Waltham, MA, USA). RNA copy values were extrapolated from the standard curve and multiplied by the volume to obtain RNA copies per organ. The limit of detection was based on the standard curve and defined as the quantity of RNA that would give a Ct value of 40.

The qRT-PCR quantification of inflammatory mediators in the lungs and brain of hamsters was performed in total RNA extracted by TRIzol reagent, as recently detailed [14].

### 2.11. Histopathology

Samples from the lungs of hamsters were fixed in formalin for 7 days and embedded in paraffin. Paraffin sections (5-µm thick) were stained with Hematoxylin and Eosin (H&E). Histopathological lesions were qualitatively described and when possible scored, using: (i) distribution qualifiers (i.e., focal, multifocal, locally extensive or diffuse), and (ii) a five-scale severity grade, i.e., 1: minimal, 2: mild, 3: moderate, 4: marked and 5: severe. In some cases, serial sections were prepared for immunohistochemistry (IHC) analyses. IHC was performed as described elsewhere [15]. Rabbit anti-N_CoV-2_ antibody (Novus Biologicals, NB100-56576) and biotinylated goat anti-rabbit Ig secondary antibody (Dako, E0432) were used in IHC. Slides were scanned using the AxioScan Z1 (Zeiss) system and images were analyzed with the Zen 2.6 software.

### 2.12. Statistical Analysis

Statistical significance was assigned when *p* values were <0.05. ELISA titers were log_10_ transformed prior to statistical analysis. For comparison of two groups, the non-parametric Mann–Whitney test was used. To compare more than 2 experimental groups, the Kruskal–Wallis test, ANOVA and Dunn’s multiple comparisons test were applied. Differences in neutralizing activity of VoCs were analyzed by two-way ANOVA with Sidak’s multiple comparisons test. Tests were performed using GraphPad Prism software (Version 9, Graphpad Software, La Jolla, CA, USA).

## 3. Results

### 3.1. Immunogenicity of LV-Encoding Various S_CoV-2_ Forms

**Figure 1:**
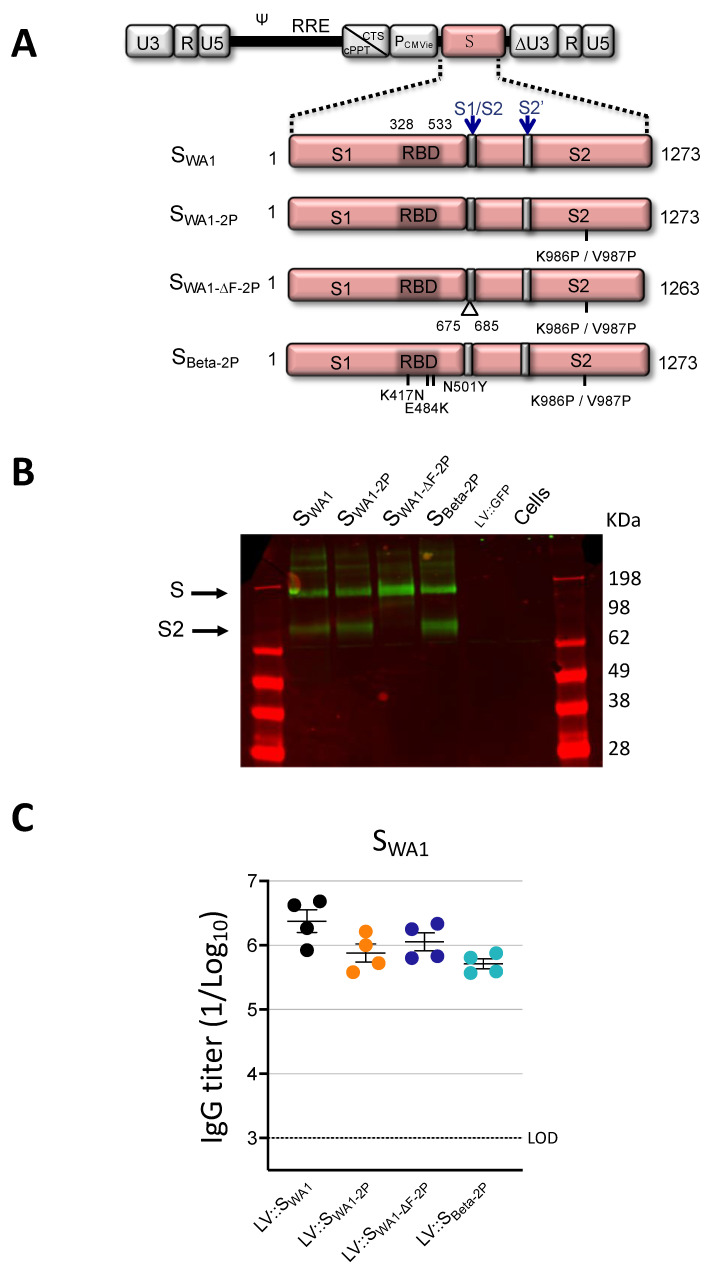
Humoral immunity in hamsters immunized i.m. with various LV::S. (**A**) Schematic representation of LV-encoding S_CoV-2_ proteins from either the ancestral WA1 or Beta SARS-CoV-2 strain. Codon-optimized sequences encoding S_CoV-2_ were cloned into the pFLAP lentiviral vector plasmid, under the control of human P_CMVie_ promoter; RRE, rev response element; cPPT, central polypurine tract. The LV::S_WA1_ includes the entire sequence of S_CoV-2_. RBD, S1/S2, S2′ cleavage sites, 675^QTQTNSPRRAR^685 sequence encompassing RRAR furin cleavage site, and K^986^P, V^987^P, K^417^N, E^484^K and N^501^Y substitutions are indicated. (**B**) Western blot analysis to detect expression of S_WA1_, S _WA1-2P_, S _WA1-ΔF-2P_, and S_Beta2-P_ in LV-transduced 293T cells. Total cell lysates were analyzed under non-reduced conditions using an anti-S2 rabbit polyclonal antibody. LV::GFP was included as negative control. Full length Spike (S) and S2 subunit are indicated. (**C**) Syrian golden hamsters (*n* = 4/group) were immunized i.m. with 1 × 10^8^ TU of LV::S_WA1_ (black circles), LV::S_WA1-2P_ (orange circles), LV::S_WA1ΔF-2P_ (blue circles) or LV::S_Beta-2P_ (green circles). Five wks later, serum anti-S_WA1_ responses expressed as the mean endpoint dilution titers, were determined by ELISA. Errors bars represent the standard error of the mean (SEM). The statistical significance of differences was determined by the Kruskal–Wallis test followed by Dunn’s multiple comparisons test and were found not significant. Dotted lines indicate the limit of detection (LOD).

Non-integrative LV-encoding stabilized conformers of S_CoV-2_ under transcriptional control of the cytomegalovirus (CMV) immediate early promoter (P_CMVie_) were constructed (Figure 1A). The first two S_CoV-2_ conformers were derived from a human codon-optimized full-length membrane anchored ancestral WA1 S_CoV-2_ [14]. LV::S_WA1-2P_ encodes a S_WA1_ which harbors two stabilizing K^986^P and V^987^P substitutions in the hinge loop of the S2 domain. LV::S_WA1ΔF-2P_ encodes a S_WA1_ which, in addition to the two K^986^P and V^987^P substitutions, is deleted of the loop encompassing the S1/S2 furin cleavage site (675-QTQTNSPRRAR-685) for further stability at the prefusion state [22,23]. S_Beta-2P_ is from the Beta (B.1.351) VoC and contains the two K^986^P and V^987^P substitutions. S_Beta_ differs from S_WA1_, notably by the N^501^Y/K^417^N/E^484^K mutations located in the RBD [24]. Whereas pseudoviruses carrying S_WA1_ were neutralized by sera from individuals vaccinated with the currently approved vaccines, those presenting these RBD mutations moderately-to-strongly resist neutralization [25]. This observation provides a rational for adapting the S sequence variant for further vaccination. Expression of S_CoV-2_ immunogens in HEK293T cells transduced with the four LV was confirmed by Western blot on total cell lysates (Figure 1B). As expected, the S2 furin cleavage product was only detected in the cells transduced by LV-encoding S_WA1_, S_WA1-2P_ or S_Beta-2P_ which harbor an intact furin cleavage site.

To compare the immunogenicity of LV::S_WA1,_ LV::S_WA1-2P,_ LV::S_WA1ΔF-2P_ and S_Beta-2P_, hamsters (*n* = 4/group) were immunized by a single i.m. injection of 1 × 10^8^ TU of either LV. Five weeks (wks) later, high serum titers of anti-S_WA1_ IgG antibodies were induced by all LV studied (Figure 1C). As no significant difference in immunogenicity between these LV was observed, LV::S_WA1ΔF-2P_, hereafter referred to as “LV::S”, was selected for evaluation of protection against homologous SARS-CoV-2.

### 3.2. Induction of Robust Humoral Responses against SARS-CoV-2 by a Single i.n. LV::S Administration

**Figure 2:**
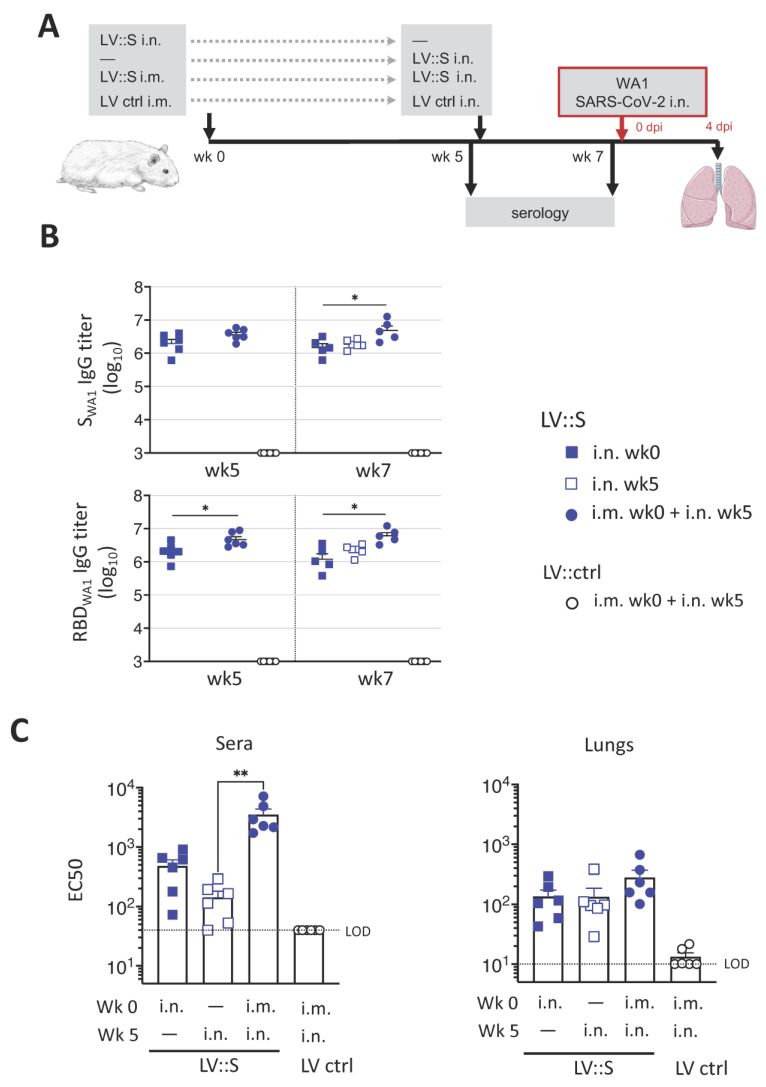
Humoral immunity in hamsters following LV::S administration. (**A**) Timeline of the LV::S prime-boost vaccination regimen and WA1 SARS-CoV-2 challenge in hamsters (*n* = 6/group). (**B**) Serum anti-S_WA1_ or -RBD_WA1_ IgG responses expressed as the mean endpoint dilution titers, determined by ELISA. (**C**) Neutralizing activity (EC50) of sera, taken prior the WA1 SARS-CoV-2 challenge, or of lung homogenates, taken at 4 dpi as determined by use of pseudoviruses harboring S_CoV-2_ from the D614G SARS-CoV-2 variant. Data are presented as the mean ± SEM. Asterisks indicate the significance of differences between the groups. *p*-values were determined by using the Kruskal–Wallis tests followed by Dunn’s multiple comparisons tests; * *p* < 0.05, ** *p* < 0.01. Only significant differences are shown. Dotted lines indicate the LOD.

We recently showed that LV::S used in a prime (i.m.)-boost (i.n.) protocol significantly improved protection against SARS-CoV-2 compared to a single i.m. injection in the hamster model [14]. Here, we evaluated the protective potential of a single i.n. administration of LV::S against the ancestral WA1 SARS-CoV-2. Hamsters (*n* = 6/group) were immunized i.n. with a single injection of 1 × 10^8^ TU of LV::S at wk 0 or at wk 5 (Figure 2A). As a positive control, a group of hamsters was primed i.m. with 1 × 10^8^ TU of LV::S at wk 0 and then boosted i.n. at wk 5 with the same amount of LV::S. Control hamsters received, following the same regimen, equivalent amounts of an LV expressing a green fluorescent protein, as an irrelevant antigen (LV ctrl). At wk 7, all animals were challenged i.n. with 0.3 × 10^5^ TCID_50_ of WA1 SARS-CoV-2 (Figure 2A). Before the challenge, pre-immune sera and those from the LV ctrl group were tested negative for anti-S_WA1_ and -RBD_WA1_ antibodies (Figure 2B). Following a single LV::S i.n. injection, all animals mounted high titers of anti-S_WA1_ and -RBD_WA1_ IgG. These antibody titers are obtained as soon as 2 wks post-immunization as shown by hamsters vaccinated at wk 5. The serum IgG titers remained stable until wk 7. At wk 7, significantly lower anti-S_WA1_ and anti-RBD_WA1_ IgG titers were detected in the groups injected i.n., compared to the i.m.-i.n. group. The sero-neutralization activity was evaluated by use of pseudoviruses harboring S_WA1_. In agreement with the anti-RBD IgG titers, sero-neutralizing activities were lower in the hamsters immunized with a single i.n. injection, compared to the i.m.-i.n. group (Figure 2C). Despite comparable anti-S and anti-RBD IgG titers at 2 or 7 wks after i.n. injection, sera from hamsters vaccinated at the earlier time point exhibited a slightly higher neutralizing capacity, suggesting the requirement of an antibody maturation over time to reach efficient neutralizing potential. However, all vaccinated groups had equivalent neutralizing capacities in their total lung homogenates, four days post-SARS-CoV-2 inoculation (4 dpi) (Figure 2C). The virus neutralizing activity in lungs can be a more relevant correlate of protection than that detectable in sera.

### 3.3. Protection against Homologous SARS-CoV-2 Challenge Induced by a Single i.n. LV::S Administration

**Figure 3:**
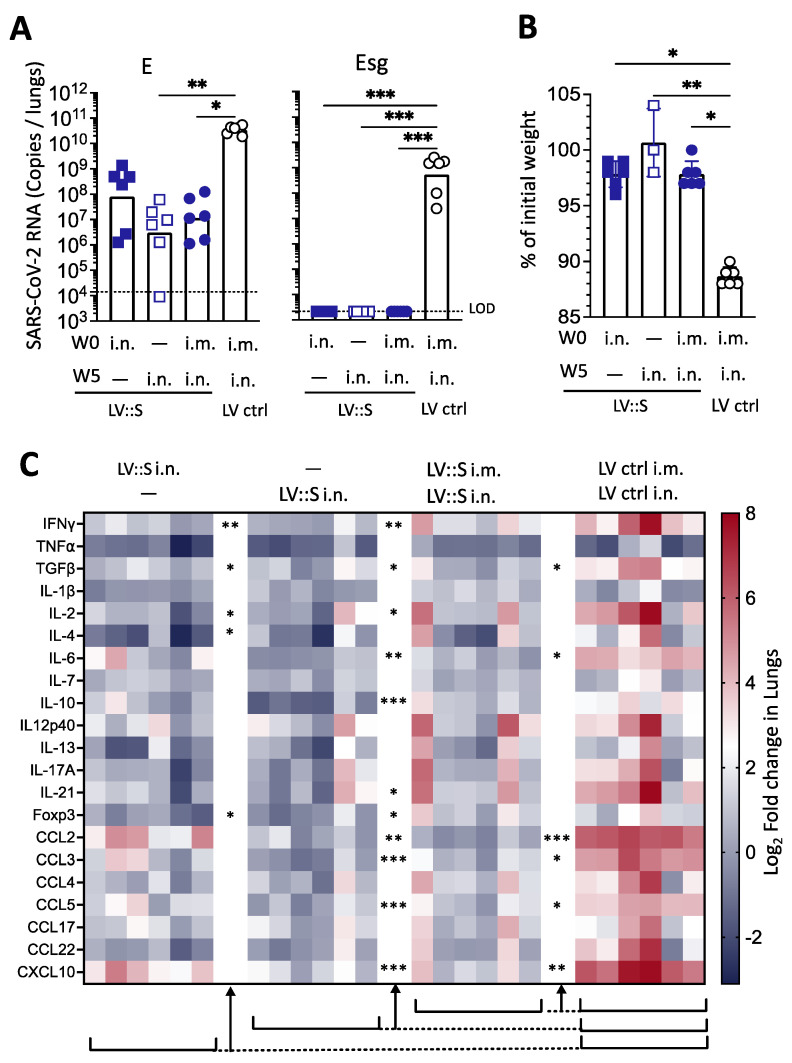
Single i.n. LV::S injection fully protects hamsters against WA1 SARS-CoV-2. Hamsters are those described in the legend to Figure 2. Hamsters were immunized either i.n. with 1 × 10^8^ TU of LV::S at wk0 (blue squares) or wk5 (open squares) or i.m. at wk0 and i.n. at wk5 (blue circles). Black circles represent hamsters injected i.m. at wk0 and i.n. at wk5 with 1 × 10^8^ TU of LV::ctrl. (**A**) Lung viral loads quantitated by total E (**left**) or Esg qRT-PCR (**right**) at 4 dpi. Bars represent geometric means. (**B**) Percentages of weight loss in LV::S- or LV ctrl-vaccinated hamsters at 4 dpi. (**C**) Expression of inflammatory cytokines in lung tissues after challenge. The heatmap recapitulates relative log_2_ fold changes in the expression of inflammation-related mediators in LV::S vaccinated or LV ctrl-administered individuals, as analyzed at 4 dpi by use of RNA extracted from total lung homogenates and normalized versus samples from untreated controls. Six individual hamsters per group are shown in the heatmap. Statistical differences between LV:: S and LV ctrl groups were determined by the Kruskal–Wallis test followed by Dunn’s multiple comparisons test and are indicated by asterisks; * *p* < 0.05; ** *p* < 0.01; *** *p* < 0.001. Comparisons were made between vaccinated groups and LV ctrl.

In the lungs of LV::S-vaccinated individuals of either i.m.-i.n. or single i.n. groups, ~2-to-4 log_10_ decreases in viral contents were observed compared to the LV ctrl group, as determined by qRT-PCR detecting the SARS-CoV-2 Envelop (E) RNA at 4 dpi (Figure 3A, left panel). Lung viral content measured by a sub-genomic E RNA (Esg) qRT-PCR is an indicator of active viral replication [21]. This analysis showed a complete absence of replicating virus in the three vaccinated groups versus a geometric mean ± SD of (5.4 ± 6.8) × 10^8^ copies of Esg RNA of SARS-CoV-2/lungs in the LV ctrl group (Figure 3A, right panel). At 4 dpi, in accordance with the protection observed, only 2–3% weight loss was detected in the hamsters vaccinated, either by i.n. alone or by i.m.-i.n. prime-boost regimen, compared to 12% weight loss in the hamsters which received LV ctrl (Figure 3B). As evaluated by qRT-PCR at 4 dpi in the total lung homogenates of the LV::S-vaccinated and SARS-CoV-2 challenged hamsters_,_ marked decreases were detected in the expression of inflammatory IFN-γ, TGF-α, IL-6 cytokines, anti-inflammatory IL-10 cytokines, and CCL2, CCL3, CCL5 and CXCL10 chemokines, as well as FoxP3, in comparison to their LV ctrl-injected and challenged counterparts (Figure 3C). Changes in inflammatory markers was particularly noticeable for the group receiving the i.n. administration 2 weeks before the challenge. In agreement with these results, a positive correlation was found between viral loads and inflammation (r = 0.46, *p* < 0.05), whereas the weights were inversely correlated with viral loads and inflammation (r = −0.6842, *p* < 0.001 and r = −0.56, *p* < 0.01, Spearman’s test), respectively.

### 3.4. Reduced Infection-Driven Inflammation in Hamsters Vaccinated with a Single i.n. LV::S Administration

**Figure 4:**
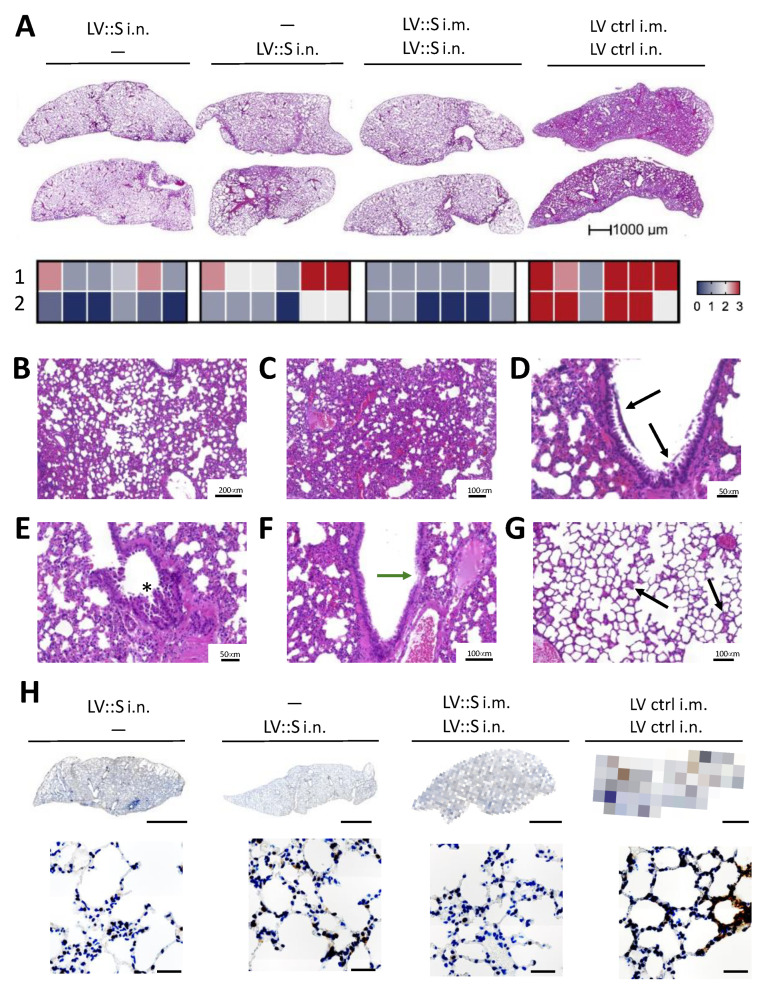
Single i.n. LV:: S injection largely reduced lung histopathogy. (**A**) Lung histological H&E analysis, as studied at 4 dpi. Heatmap recapitulating the histological scores, for: (1) inflammation score and (2) interstitial syndrome. (**B**) Representative alveolo-interstitial syndrome and (**C**) severe inflammation in an LV ctrl-injected and infected hamster. Here, the structure of the organ is largely obliterated, while remnants of alveolar spaces and bronchiolar lumens can be seen. (**D**–**F**) bronchiolar lesions in LV ctrl-immunized animals. Shown are epithelial cells and cell debris in the bronchiolar lumen (black arrows) (**D**), papillary projections of the bronchiolar epithelium into the lumen (star) (**E**) and degenerative lesions with effacement of the epithelium (green arrow) (**F**). (**G**) Mild alveolar infiltration in a vaccinated hamster. Some of the alveoli (arrow) are partially or completely filled with cells and an eosinophilic exudate. (**H**) Representative N_CoV-2_-specific IHC image performed on lungs of SARS-CoV-2-infected hamsters. Lower panels show enlarged views from upper panels. Scale bars are 1 mm for upper panels and 25 μm for lower panels.

On lung histopathological examination, vaccinated controls demonstrated lung infiltration (Figure 4A) and severe alveolo-interstitial inflammation (Figure 4B) leading to dense pre-consolidation areas (Figure 4C). These lungs also displayed bronchiolar lesions, with images of epithelial sloughing of individual or clustered cells (Figure 4D) and of hyperplastic epithelial growth producing papillary projections (Figure 4E) or intraluminal epithelial folds (Figure 4F). In vaccinated groups, the interstitial (Figure 4A) and alveolar (Figure 4G) lesions were minimal to moderate. Immunohistochemistry analysis of the lungs of LV ctrl-treated and infected hamsters, with a SARS-CoV-2 nucleocapsid protein (N_CoV-2_)-specific polyclonal antibody, detected numerous clusters of N_CoV-2_^+^ cells in the bronchial epithelial cells (not shown) and in the interstitium (Figure 4H, right panels). In contrast, the severity of inflammation was reduced in LV::S-vaccinated animals. When detectable, the inflamed zones still contained N_CoV-2_^+^ cells, indicating that, although viral replication has been controlled (Figure 3A), infiltration and virus remnants have not yet been fully resorbed at the early 4 dpi time point.

These data collectively indicated that immunization with a single i.n. administration of LV::S was as efficient as an i.m. prime followed by a i.n. boost regimen and conferred strong protective immunity against an homologous SARS-CoV-2 infection.

### 3.5. LV::S_Beta-2P_ Prime (i.m.)-Boost (i.n.) Vaccination Cross-Protects against Omicron Variant

**Figure 5:**
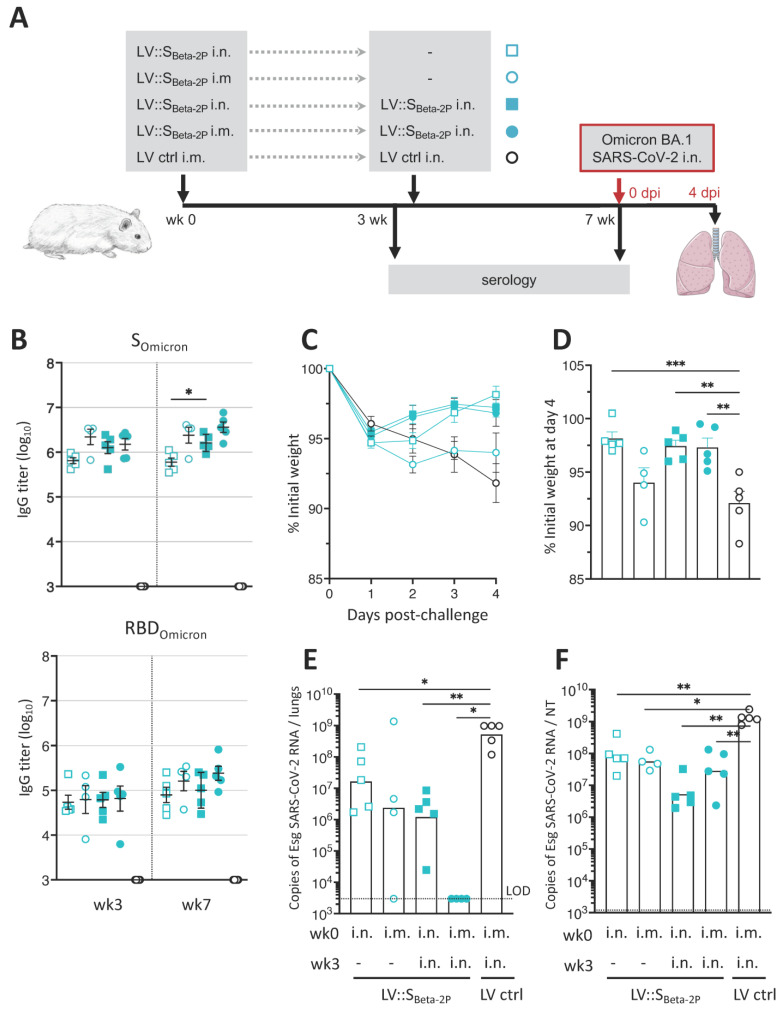
Decreased SARSCoV-2 omicron infectious virus in lungs and nasal turbinates by i.n administration of a single or booster dose of LV::S_Beta-2P_. (**A**) Timeline of single or prime-boost vaccination and Omicron SARS-CoV-2 challenge. Hamsters (*n* = 4–5/group) were primed i.n. (squares)or i.m. (circles) with 1 × 10^8^ TU of LV::S_Beta-2P_. Three weeks later, some of them were boosted i.n. with the same amount of LV::S_Beta-2P_ (filled symbols) or LV ctrl (black circles). Serum samples were collected at wks 3 and 7 for serological analyses. (**B**) Serum or anti-S_Omicron_ (upper panels) or -RBD_Omicron_ (lower panels) IgG responses, expressed as the mean endpoint dilution titers, determined by ELISA. Data are presented as the mean ± SEM. Percentages of weight loss in LV::S_Beta-2P_-, or LV ctrl-vaccinated hamsters following challenge (**C**) and at 4 dpi (**D**). (**E**) Lung and (**F**) NT viral loads were quantitated by Esg qRT-PCR at 4 dpi. Bars represent geometric means. Statistical differences were determined by the Kruskal–Wallis test followed by Dunn’s multiple comparisons test and are indicated by asterisks. * *p* < 0.05, ** *p* < 0.01, *** *p* < 0.001. Dotted lines indicate the LOD.

Given the dynamics of the pandemic, an important question is the ability of vaccines to induce cross-protection against new VoCs. Based on a series of LVs encoding for S from various VoCs, we recently selected LV::S_Beta-2P_ as the best candidate to generate the broadest spectrum of cross-neutralization potential [26]. To evaluate the efficacy of LV::S_Beta-2P_ in the hamster model against SARS-CoV-2 Omicron, hamsters (*n* = 4–5/group) were primed i.m. or i.n. at wk0 with 1 × 10^8^ TU of LV::S_Beta-2P_. At wk3, one group of each were boosted i.n. with the same dose of LV::S_Beta-2P_ (Figure 5A). All groups were challenged at wk7 with 0.3 × 10^5^ TCID_50_ of SARS-CoV-2 BA.1 Omicron sub-variant [19]. S_Omicron_ harbors 32 mutations compared to S_WA1_. Among these mutations,15 are located in the RBD. Infection of hamsters with this BA.1 Omicron strain, isolated from a patient, resulted in a significant decrease in weight (Figure 5B).

Robust cross-reactive serum IgG titers were detected by ELISA against S_Omicron_ and RBD_Omicron_ in all LV::S_Beta-2P_-vaccinated hamsters (Figure 5B). No significant differences in antibody titers between the groups were observed 3wks post-prime. Antibody levels remained stable after the single injections, while a significant increase in anti-S_Omicron_ titers was observed in the animals primed and boosted i.n.. By contrast, anti-RBD antibody titers continued to rise in all vaccinated groups over time (Figure 5B, lower panel).

Following challenge, hamsters vaccinated by a single i.m. injection of LV::S_Beta-2P_ or those who received LV ctrl gradually lost weight (Figure 5C). Hamsters vaccinated i.n. with LV::S_Beta-2P_ exhibited less than 5% of weight loss, without signs of morbidity (Figure 5D). At 4 dpi, viral contents in the lungs and in nasal turbinates were analyzed. High viral contents were detected in both organs of the LV ctrl-injected group (Figure 5E,F). In contrast, no Esg RNA was detected from the lungs of the i.m.-i.n. group and significant reductions of ~2 log were observed in the other groups (Figure 5E). Of note, the i.m. vaccinated hamster which did not control viral replication had the highest weight loss. Although also significantly reduced, active viral replication was still detectable in the NT of all hamsters, indicating that LV-based i.n. vaccination, despite its strong efficacy in the protection of the lungs, does not fully prevent nasal infection (Figure 5F). However, an i.n. boost, regardless of the route of prime, led to a better efficacy over a single vaccine administration in the control and the spread of infection in the respiratory tract tissues.

### 3.6. Decrease in Virus Content as Determined by Immunohistochemistry in LV:: S_Beta-2P_-Vaccinated Hamsters

**Figure 6:**
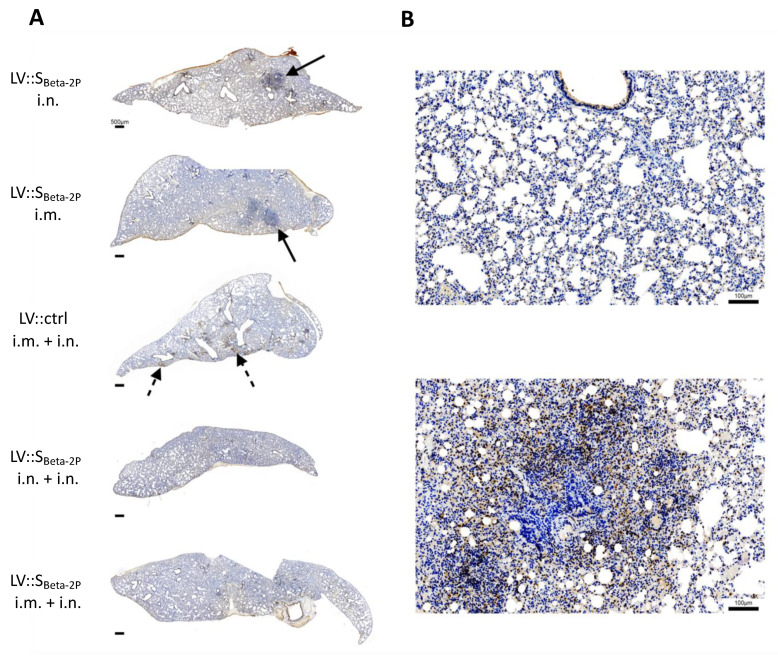
Immunodetection of the NCoV-2 antigen performed on lungs of Omicron SARS-CoV-2-infected hamsters. Hamsters are those described in Figure 5. (**A**) One example of each vaccination regimen is shown at low magnification. Solid arrows denote foci of inflammatory infiltrates, and dotted arrows areas where the immunodetection signal is discernable even at this low magnification. (**B**) The close-up views depict the concentration of viral antigen (brown) within the inflammatory foci (**bottom**), while areas harboring no or little inflammation (**top**) display only scarce staining.

At 4 dpi, histopathological analysis of the lung sections in the ctrl group showed similar lesions detailed in Figure 4H (Figure 6). Immunohistochemistry images displayed a generally less abundant N_CoV-2_ staining in mice boosted i.n or i.m, relative to the primed-only and LV ctrl-injected animals, although there was a relatively high degree of intra-group variation (Figure 6). In addition, we did not observe a tight correlation between the extent of the IHC signal and the Esg qRT-PCR quantifications, indicating that part of the immunostained antigen corresponds to non-replicating virus remnants.

Altogether, these results showed that while a single i.n. immunization with LV can be enough to control the infection, an LV-based i.n. administration will be better adapted to boost a previously induced anti-COVID-19 immunity.

### 3.7. Induction of Cross-Reactive Antibodies in LV::S_WA1-2P_-Primed and LV::S_Beta-2P_-Boosted Hamsters

**Figure 7:**
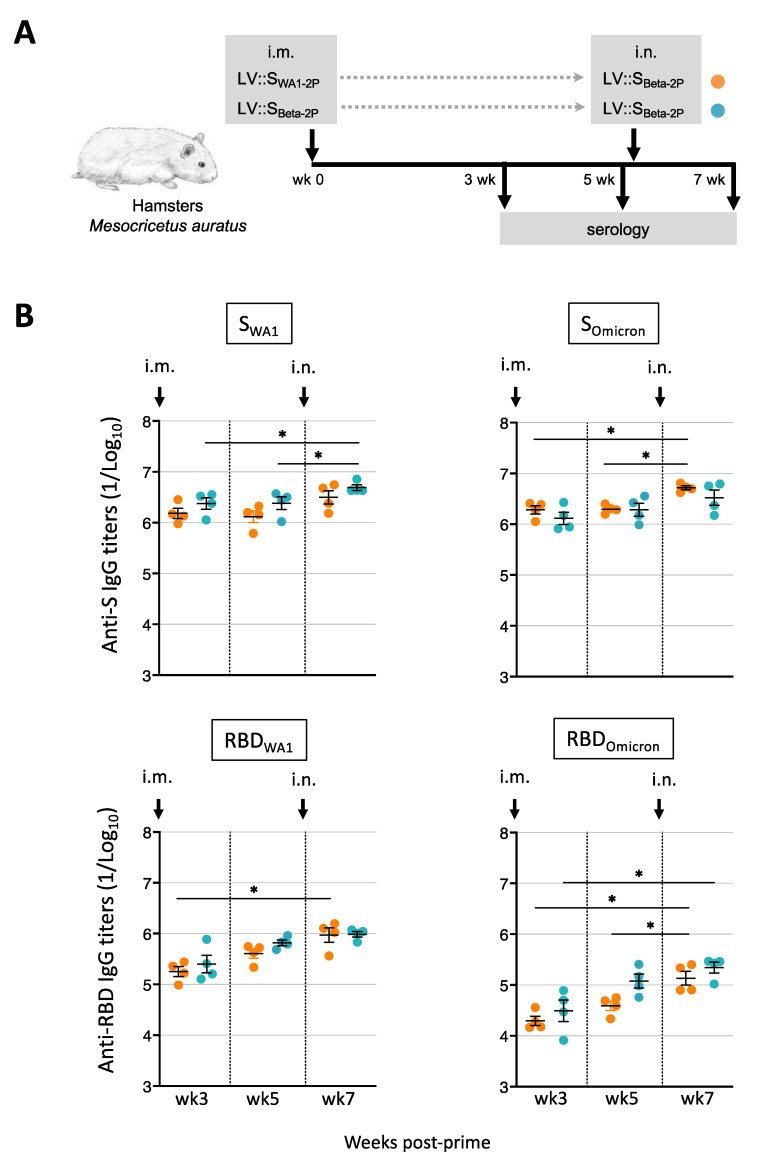
Robust humoral responses in hamsters vaccinated by LV::S_WA1-2P_ or LV::S_Beta-2P_ prime (i.m.)-LV::S_Beta-2P_ boost (i.n.). (**A**) Timeline of prime-boost vaccination. Hamsters (*n* = 4/group) were primed i.m. with 1 × 10^8^ TU of LV::S_WA1-2P_ or LV::S_Beta-2P_. Five weeks later, hamsters were boosted i.n. with the same amount of LV::S_Beta-2P_. (**B**) Serum anti-S_WA1_ or -RBD_WA1_ (left panels) or anti-S_Omicron_ or -RBD_Omicron_ (right panels) IgG responses, expressed as the mean endpoint dilution titers ± SEM, determined by ELISA. Statistical differences are indicated by asterisks. * *p* < 0.05.

We then evaluated the efficacy of an LV::S_Beta-2P_ i.n. boost in animals previously exposed to S_WA1_. Hamsters (*n* = 4/group) were primed i.m. at wk0 with 1 × 10^8^ TU of LV::S_WA1-2P_ or LV::S_Beta-2P_. At wk5, both groups were boosted i.n. with 1 × 10^8^ TU of LV::S_Beta-2P_ (Figure 7A). Robust serum IgG titers were detected against S and RBD proteins at any post-prime time point tested, in all vaccinated hamsters (Figure 7B). After the prime or after the boost, comparable kinetic profiles and intensities of S_WA1_- or S_Omicron_-specific antibody responses were observed (Figure 7B, upper panels). Either the homologous or the heterologous i.n. boost marginally increased the anti-S antibody titers by 1.8- or 2.5-fold, respectively. By contrast, ~4- to 10-fold lower serum IgG responses against RBD_Omicron_ were measured compared to RBD_WA1_ (Figure 7B, lower panels). However, anti-RBD_Omicron_ IgG titers were significantly better improved by heterologous boost than by homologous boost with a 3.8- versus 1.7-fold increase, respectively.

### 3.8. Anti-S_CoV-2_ Antibody Imprinting in LV::S-Primed and LV::S_Beta-2P_-Boosted Hamsters

**Figure 8:**
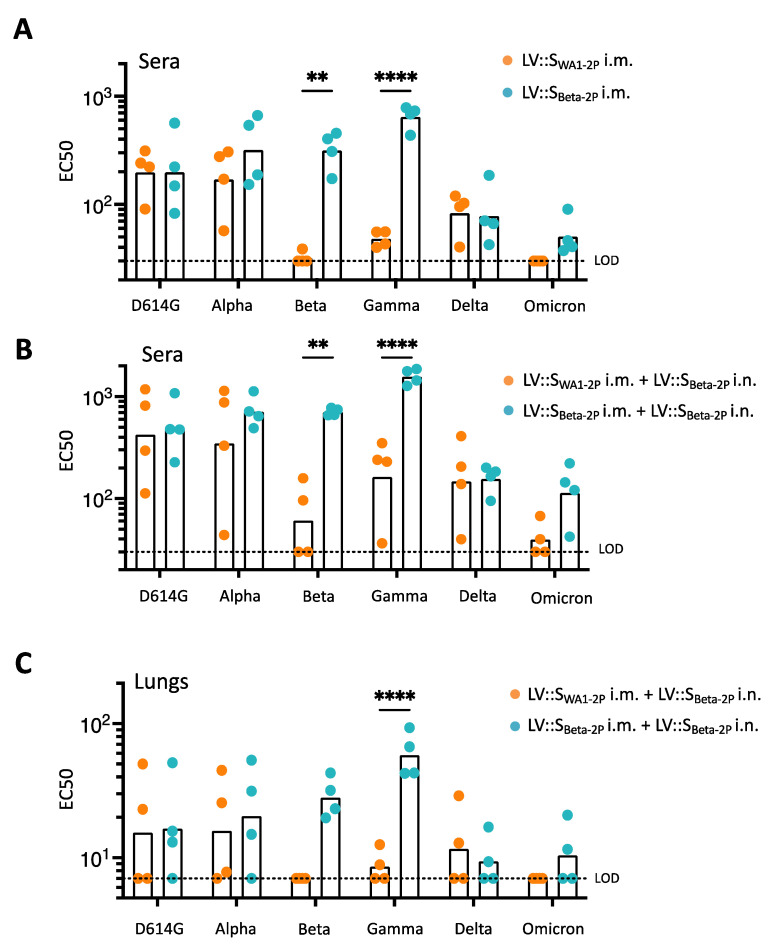
Anti-S_CoV-2_ antibody imprinting in hamsters vaccinated by LV::S prime (i.m.)-LV::S_Beta-2P_ boost (i.n.). Hamsters are those described in the legend to Figure 7. (**A**–**C**) EC50 determined by use of pseudo-viruses carrying S_CoV-2_ from the D614G, Alpha, Beta, Gamma, Delta or Omicron variants. EC50 (**A**) 5 wks post-prime in sera or 2 wks post-boost in sera (**B**) and in lung homogenates (**C**). Data are expressed as the geometric mean EC50. Statistical significances were analyzed using two-way ANOVA followed by Sidak’s multiple comparisons test; ** *p* < 0.01; **** *p* < 0.0001. Dotted lines indicate the lower limit of detection (LOD).

Five wks post-i.m. injection, both LV::S_WA1-2P_ and LV::S_Beta-2P_ induced high sero-neutralizing activities against pseudoviruses harboring S_D614G_ or S_Alpha_ (Figure 8A). Cross-neutralizing activity against S_D614G_, S_Alpha,_ and S_Delta_ was similar in the two groups of immunized hamsters. Of note, after a single i.m. injection, only LV::S_Beta-2P_-immunized hamsters exhibited sero-neutralization activity against all S variants, although weaker against S_Omicron_.

LV::S_Beta-2P_ i.n. boost increased the cross sero-neutralization potential against all VoCs in both groups (Figure 8B). Although the levels of neutralizing antibodies were improved in the sera from the LV::S_WA1-2P_-primed and LV::S_Beta-2P_-boosted hamsters, they were barely able to cross-neutralize pseudoviruses harboring S_Beta_ and totally unable to cross-neutralize pseudoviruses harboring S_Omicron_ (Figure 8B). Lung homogenates exhibited a similar profile with no cross-neutralizing activities against S_Beta_ or S_Omicron_ following the heterologous prime-boost (Figure 8C). In net contrast, sera and lung homogenates from LV::S_Beta-2P_-primed and LV::S_Beta-2P_-boosted hamsters were much better able to cross-neutralize pseudoviruses harboring S_Beta_ or S_Gamma_ and—to a lesser extent—those harboring S_Omicron_. However, this prime-boost regimen was sufficient to confer protection against SARS-CoV-2_Omicron_ challenge, as observed above (Figure 5). Therefore, an LV::S_Beta-2P_ boost improves cross sero-neutralization much better in LV::S_Beta-2P_-primed hamsters than in their LV::S_WA1_-primed counterparts. These results show a clear imprinting effect in anti-S humoral immunity, and particularly obvious against the Omicron and Beta variants.

## 4. Discussion

The LV-based platform has emerged recently as a powerful vaccination approach against COVID-19. We notably demonstrated its strong prophylactic capacity at inducing protection in the lungs against SARS-CoV-2 infection when used as a systemic prime followed by mucosal i.n. boost [14]. In the present study, as a further step toward a clinical trial, we used LV-encoding stabilized forms of S_WA1_ or S_Beta_. This choice was based on data indicating that stabilization of viral envelop glycoproteins in their prefusion forms improves the yield of their production as recombinant proteins in industrial manufacturing of subunit vaccines. Moreover, it also increases the efficacy of nucleic acid-based vaccines, by raising availability of the antigen under its optimal immunogenic shape [27].

In the first part of this report, we demonstrated that a single i.n. administration of an LV-encoding S_WA1_ confers, as efficiently as an i.m.-i.n. prime-boost regimen, full protection of lungs in the highly susceptible hamster model, as evaluated by virological, immunological and histopathological parameters. The hamster ACE2 ortholog protein interacts efficaciously with S_CoV-2_, readily allowing host cell invasion by SARS-CoV-2 with high replication rates. With rapid weight loss and development of severe lung pathology subsequent to SARS-CoV-2 inoculation, outbred hamsters provide a sensitive model to evaluate the efficacy of drug or vaccine candidates [28]. Hamsters represent a more challenging model than Rhesus macaques, which develop only a mild COVID-19 pathology. The strong protection of the lung conferred by a single i.n. administration against homologous challenge in the hamster model is therefore an asset of primary importance. This protection most likely results from the development of a mucosal immunity. Induction of antigen-specific secretory dimeric IgA that can block the interaction of the virus at the mucosal level have been shown to reduce the viral shedding and to correlate with protection [29,30,31]. Although infectious virus was still detected in the nasal turbinates of i.n.-immunized hamsters, the significant reduction in infectious viral titers could lead to reduced transmission and dissemination as recently described by Langel SN et al. providing a means of disease control [32]. Indeed, we have previously shown in the mouse model that anti-S IgG and secretory IgA antibodies were generated in lungs, together with lung resident memory B and T cells following i.n. LV administration. The presence of IgA induced by LV-based SARS-CoV-2 vaccines correlates with complete pulmonary protection against the virus [26]. Unfortunately, the mucosal immunity could not be assessed in the hamster model because of the lack of immunological tools, including anti-IgA antibodies and antibodies to activation/memory T-cell markers. Meanwhile, there is growing evidence that i.n. immunization provides a better protection, not only against the SARS-CoV-2 ancestral strain, but also against newly emerged VoCs [33,34]. The studies exploring this domain so far used chimpanzee adenoviral vectored vaccines that are known to be pro-inflammatory, and thus risky for use in mucosal vaccination [35]. In net contrast, LVs are non-cytopathic and very weakly inflammatory [10] and much more suitable for mucosal vaccination. The fact that a single i.n. LV-based vaccine administration, either 2 or 7 wks before homologous SARS-CoV-2 challenge, elicits protection is valuable in setting clinical trials with LV-based vaccines. This platform can provide remarkable advantages for mass vaccination, with the major advantage of mucosal immunization in the reduction in viral transmission.

The continued emergence of SARS-CoV-2 VoCs prompted us to expand our study by assessing the protective potential of a heterologous antigen booster which could, in terms of anti-S antibody response, mimic some aspects of a previous infection or earlier vaccination with the first-generation vaccines, mainly based on S_WA1_. Numerous breakthrough SARS-CoV-2 infections have been observed in vaccinated individuals, showing the incomplete cross-efficacy of these vaccines [36,37]. Recently, it has been reported that mucosal booster vaccination is needed to establish robust sterilizing immunity in the respiratory tract against SARS-CoV-2 [38]. In LV-immunized hamsters, we did not detect striking differences between the ability of S_WA1_ and S_Beta-2P_ antigens to induce cross-reactive serum IgG responses against S_CoV-2_. However, a clear distinction should be made between the protective capacity of vaccines and their ability to induce neutralizing antibodies, since T-cell responses are also major effector players against SARS-CoV-2 infection. In particular, the effectiveness of LV-based protection is not only dependent on the capacity to induce neutralizing antibody responses but also, and to a large extent, on their T-cell immunogenicity. It is noteworthy that an almost complete protection of lungs is achieved in µMT KO mice that are totally devoid of mature B-cell compartment and antibody response [15]. In addition, mucosal resident memory T cells, as well as IFNγ^+^ IL-2^+^ TNF^+^ triple positive CD8^+^ T cell effectors, are readily detectable in the lung of LV::S-primed (i.m.) and boosted (i.n.) mice [26]. Furthermore, findings obtained following natural infection largely suggest that specific T-cell immunity, which is generally less affected by mutations occurring in the S antigen of emerging SARS-CoV-2 variants, are largely effective against viral replication [39,40]. T-cell mediated protection is also certainly operating in hamsters. However, as mentioned above, the lack of immunological tools prevented the characterization of T-cell responses in the present study.

In the LV::S-primed and LV::S_Beta-2P_-boosted hamsters, despite the enhanced sero-neutralizing potential against D614G, Alpha and Delta variants, largely statistically reduced cross-neutralization activities were observed against Beta and Gamma variants and no cross-neutralization activity was observed against the Omicron variant. Likewise, in the cases of influenza A viruses, a first exposure to a serotype can affect future responses to its variants [41]. This raises concerns about immune imprinting effects of previous infections or vaccinations on antibody responses, which will need to be considered when designing vaccines [42]. The present study indicates that pre-exposure of the immune system to an early S variant has a negative impact on the neutralizing antibody response, measured after a late booster with a heterologous S variant. Our results corroborate recent data showing that healthcare workers infected either by SARS-CoV-2 ancestral or Alpha variant exhibit a reduced neutralizing immunity against Omicron [43]. Moreover, using mRNA vaccines, Kalnin et al. also showed that heterologous boosting provided inferior neutralizing antibody titers compared to homologous boosting [44]. The hypothesis can be put forward that additional injections of the variant S sequence could be required to counteract this negative effect and to reach sufficient levels of cross-neutralization against VoCs.

Collectively, our results demonstrate the ability of the LV as an effective vaccine delivery platform. LV is an effective and promising strategy to elicit a strong protective immunity against SARS-CoV-2 VoCs and possesses the advantage to be non-inflammatory and thus suitable for use in mucosal i.n. vaccination. We have recently demonstrated the safety of LV::S_Beta-2P_ i.n. administration in mice in which the high dose of 1 × 10^9^ TU of LV had been injected [26]. No adverse effects had been detected by lung histopathological analyses. During the transition to the preclinical phase, other possible safety concerns, such as inflammation in nasal cavity and possible transport to the brain via olfactory nerves, will obviously be included in the follow-up points.

## 5. Patents

BV, PA, IF, AN, MWN, LM, MB and PC are inventors of patents either published or pending, directed to the potential of i.n. LV::S_CoV-2_ vaccination.

## Data Availability

The datasets generated during and/or analyzed during the current study are available from the corresponding author, M.B., on reasonable request.
